# 3D Printing of Amino Resin-based Photosensitive Materials on Multi-parameter Optimization Design for Vascular Engineering Applications

**DOI:** 10.3390/polym11091394

**Published:** 2019-08-24

**Authors:** Yung-Cheng Chiu, Yu-Fang Shen, Alvin Kai-Xing Lee, Shu-Hsien Lin, Yu-Chen Wu, Yi-Wen Chen

**Affiliations:** 1 School of Medicine, China Medical University, Taichung 40447, Taiwan; 2 Department of Orthopedic Surgery, China Medical University Hospital, Taichung 40447, Taiwan; 3 Department of Bioinformatics and Medical Engineering, Asia University, Taichung 40447, Taiwan; 4 3D Printing Medical Research Institute, Asia University, Taichung 40447, Taiwan; 5 3D Printing Medical Research Center, China Medical University Hospital, Taichung 40447, Taiwan; 6 Graduate Institute of Biomedical Sciences, China Medical University, Taichung 40447, Taiwan

**Keywords:** tissue engineering, blood vascular graft, DLP Technology, design of experiments, amino resin, dopamine

## Abstract

Cardiovascular diseases are currently the most common cause of death globally and of which, the golden treatment method for severe cardiovascular diseases or coronary artery diseases are implantations of synthetic vascular grafts. However, such grafts often come with rejections and hypersensitivity reactions. With the emergence of regenerative medicine, researchers are now trying to explore alternative ways to produce grafts that are less likely to induce immunological reactions in patients. The main goal of such studies is to produce biocompatible artificial vascular grafts with the capability of allowing cellular adhesion and cellular proliferation for tissues regeneration. The Design of Experimental concepts is employed into the manufacturing process of digital light processing (DLP) 3D printing technology to explore near-optimal processing parameters to produce artificial vascular grafts with vascular characteristics that are close to native vessels by assessing for the cause and effect relationships between different ratios of amino resin (AR), 2-hydroxyethyl methacrylate (HEMA), dopamine, and curing durations. We found that with proper optimization of fabrication procedures and ratios of materials, we are able to successfully fabricate vascular grafts with good printing resolutions. These had similar physical properties to native vessels and were able to support cellular adhesion and proliferation. This study could support future studies in exploring near-optimal processes for fabrication of artificial vascular grafts that could be adapted into clinical applications.

## 1. Introduction

Cardiovascular disease is a common disease worldwide and many scientists have put in great efforts to explore the best treatment methodology for cardiovascular diseases. It is currently estimated that there are up to 15 million deaths worldwide from cardiovascular diseases annually [1,2,3]. The artificial vascular grafts that are available in the market now are mainly made up of Dacron and polytetrafluoroethylene (ePTFE) and such grafts often have a diameter of more than 6 mm as the characteristics of Dacron make it unsuitable to fabricate grafts with smaller diameters [4,5]. In addition, Dacron has poor biocompatibility which limits its regeneration capability after transplantation. On the other hand, artificial grafts comprising of ePTFE have good biocompatibility and good physical properties that allowed surgeons to have more room for surgical procedures. Furthermore, small pores to be fabricated using ePTFE and one of the main advantages of ePTFE grafts is that it does not require any pre-anti-coagulant before implantations. However, it was reported that the wall of the grafts was more prone to pseudoaneurysm and it has little longitudinal elasticity, thus restricting seeding of endothelial cells [6,7]. Therefore, the aim of this study was to develop artificial vascular grafts with structures similar to native blood vessels with the final goal of achieving regenerative medicine and organ reconstruction standards [8,9].

Since the past decades, tissue engineering had evolved tremendously and one of the recent focus of tissue engineering was on developing artificial biological organs substitutes either for transplantations or as models for drug screening [10,11]. The greatest feature of tissue engineering is in its inherent biological stability, which is characterized by successful implantation and healing [12,13]. There are three key elements in tissue engineering and these elements include cells, scaffolds and environmental factors [14]. In the early years, scaffolds were thought to be only providing cells with temporary support for cell growth [15]. However, modern scaffolds have evolved to be a critical factor in tissue engineering and scaffolds were reported to be more than a mere container or support [16]. Scaffolds are thought to resemble the “extra-cellular matrix” of native micro-environment and certain structural properties of scaffolds are reported to be able to influence cellular differentiation into certain cell lines by providing biological, chemical and mechanical cues [17,18]. In this study, we aimed to develop scaffolds for vascular tissue engineering and hoped that such a study can be used as a platform for potential vascular implantations.

Currently, 3D printing is considered as an effective and potential technology to revolutionize the field of regenerative medicine and tissue engineering [19,20]. Recently, a novel multi-layered manufacturing concept had emerged and had since garnered attention as it was able to provide answers to problems that were unable to be solved previously and thus this concept increases the possibility of cure for many diseases [21,22]. Additive manufacturing is a very flexible technology and as proven, it can be replaced with biomedical materials combined with tissue engineering to fabricate biocompatible scaffolds for more medical research [23]. The combination of additive manufacturing and tissue engineering has made it possible to create more complexed scaffolds with highly mimicry structures as native tissues [20,24,25].

In recent years, different types of 3D printing techniques had emerged and it includes fused deposition modeling (FDM), selective laser sintering (SLS), stereolithography (SLA) and digital light processing (DLP) [24,26,27]. Each technology has its own advantages and disadvantages and it was reported that DLP technology had the potential to print with high printing resolutions thus we were able to fabricate scaffolds with small pores. However, a key component of DLP technology lies in the chosen material [28]. Currently, there are numerous types of printable materials in the market, however, it is important to note that biological or implantable scaffolds require certain unique characteristics such as biocompatibility, degradability and ample mechanical properties. In addition, printing parameters also have a role to play in determining the performance and biocompatibility of the printed product. In the past, most research was focused on material development and scaffold printings and was less focused on printing parameters and 3D printer printing settings. Therefore, the aim of this study was not only to develop biocompatible photopolymerization curing materials but also looked into the fine-tuning of 3D printer printing setting parameters as one of the studies focus into the design and fabrication of artificial vascular grafts. There were current report studies that used indirect manufacturing methods with 3D printing technology to fabricate channel molds to develop artificial vascular grafts [29]. However, this indirect method requires longer manufacturing time and such grafts were still found to be different as native vessels. In 2012, Meyer et al. used DLP to print soft artificial blood vessels and assessed for its physical properties. However, the vascular grafts were not structurally and strategically designed. Current studies had shown that different structural supports and designs can help in promoting cellular adhesion, proliferation, and differentiation. In addition, biocompatibility tests were not conducted for the study conducted by Meyer et al. thus casting doubts regarding their fabricated graft and cellular support [30]. In 2016, Melchiorri et al. used DLP 3D printing technology to print elastic artificial vascular grafts and implant them into related animal experiments [31]. Similarly, the grafts that they fabricated had no proper structural designs. A recent study by Kroll and Buchris used DLP 3D printing technology to print soft artificial vascular grafts with pores, but it was unfortunate that degradation studies and profiling were not conducted in their study [32]. Single layered vascular grafts with pores may cause pre-mature degradation and thus leading to blood leakage as new vascular tissues had not yet been established. Furthermore, native blood vessels have three or four differing layers thus the studies above were not able to mimic the multi-layered characteristics of native vessels. Therefore, this study aimed to solve the shortfalls from previous studies and attempt to develop vascular grafts with similar biological, physical properties and functions of native blood vessels so as to further aid the development of vascular tissue engineering [33,34].

There are several factors to consider when developing an artificial vascular graft which included materials, printing processes, and post-treatments. In this study, we used a photocurable amine-based resin (AR) as the printing material with a natural material dopamine (DA) as a light absorber to improve the printing resolution. In addition, statistical design of experiments (DoE), which is the science of obtaining the largest possible amount of information about a system with the smallest number of experiments, was also applied to this research [35]. For the experimental designs, we conducted 23 full factor experiments and added three center points for each study. The selected testing factors were ratio of materials, addition of dopamine and printer setting parameters. In this study, we developed biomedical materials that can be applied to DLP 3D printing technology and further used this material in combination with bionic artificial vascular graft designs to achieve high accuracy, biocompatibility and physical properties that were similar with native vascular properties, including strength, elasticity, and porosity. Furthermore, by attempting to determine optimal parameters for fabrication of vascular grafts, we could pave an effective method for future tissue reconstruction studies and also facilitate the long-term goal of developing a suitable and implantable vascular graft.

## 2. Materials and Methods

### 2.1. Experimental Design

Experimental design and statistical evaluation can ease, as well as improve the property of experimentation, by evaluating and recognizing the most important parameters and interactions of such parameters that are likely to occur [36]. Resolution statistical design was applied for DA-contained AR materials formulation to consider the effect of formulation processes and parameters (such as stirring speed and rate, injection volume and concentrations of the used excipients) in two different extreme levels (Table 1). The significant effect of these formulation independent variables in Table 1 were examined on the dependent variables viz. resolution (response 1), Young’s modulus (response 2), and cell viability (response 3), using Design-Expert®. The design generated 11 experiments as shown in Table 2.

### 2.2. Preparation of Amino Resin-based Composites

50% AR AgiSyn 003 (DSM-AGI, Nederland) was placed into Tris-(hydroxymethyl) aminomethane (Tris, Sigma-Aldrich, MO, USA) buffer (10 mM, pH 8.5) with 0.1–3 mg/mL dopamine (DA, Acros Organics, NJ, USA) and 1.2 mg/ml of ammonium persulfate (Sigma-Aldrich, St. Louis, MO, USA) added next. The AR-based composites were mixed using the ultrasonic oscillator for 5 min, after which the composites turned dark brown after mixing. The hybrid materials were then heated at 180 °C and stirred to dehydrate the mixtures. Various amounts of 1.5% 2,4,6-trimethylbenzoyl-diphenyl-phosphineoxide (TPO, Ciba Specialty Chemicals Inc., Basel, Switzerland) photo-initiators, 0.1% 2-Hydroxy-4-methoxybenzophenone-5-sulfonic acid (HMBS, Tokyo Chemical Industry, Tokyo, Japan) and 2,2,6,6-Tetramethylpiperidine-1-oxyl (TEMPO, Acros Organics, Morris Plains, NJ, USA) were dissolved in 2-Hydroxylethyl methacrylate (HEMA, Sigma-Aldrich and then added to the AR-based composites, mixed at 80 °C to form the 40–80% AR-contained resin for 3D printing.

### 2.3. Artificial Blood Vessel Fabrication

**Figure 1:**
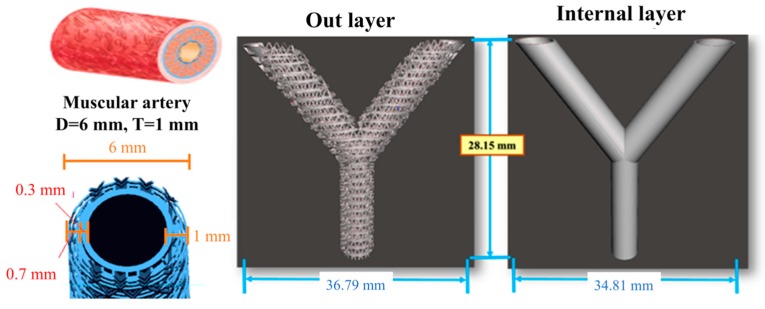
The design of the bi-layer engineering blood vessel scaffold.

All test objects and scaffolds were designed using SolidWorks (Dassault Systemes SolidWorks Corp., MA, USA) and fabricated with a MiiCraft high resolution DLP 3D printer (Young Optics Inc., Hsinchu, Taiwan). Figure 1 showed design of vascular grafts with triangular holes which was previously reported to facilitate cell growth. The diameter of the vascular graft was determined to be at 6 mm and this decision was with reference to the diameter of human muscular artery. In addition, the thickness of the wall was determined to be at 1 mm. For printing, an individual 100 μm layer was exposed to blue light (405 nm) digital stereolithography for 23 to 40 s depending on the concentrations of the DA-contained AR resins. The uncured materials were washed off and the scaffolds were post-cured under UV light, yielding the fully cured scaffolds. The cured scaffolds were washed again for cell culture.

### 2.4. Printing Accuracy Analysis

**Figure 2:**
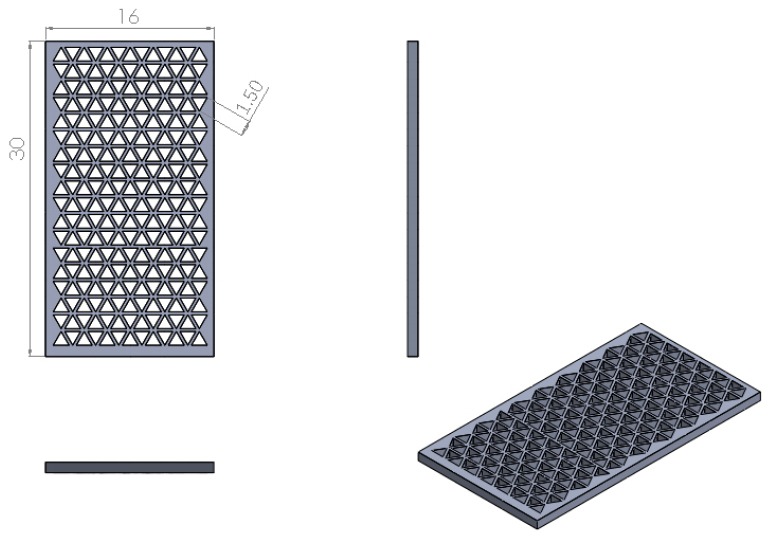
The design of resolution test specimens.

Figure 2 showed the samples which were designed for printing accuracy analysis. The internal dimensions of the test piece were determined to have a length of 30 mm and a width of 16 mm to produce equilateral triangles. In addition, the length of the regular triangle was designed to be at 1.5 mm, with the total quantities set at a maximum of 234 equilateral triangles. Briefly, a digital microscope was used to use to capture images of the scaffolds and analyzed using the MATLAB software (MathWorks, Natick, MA, USA). Pixel sizes with a scale of 5 mm were calculated using the program and the area of an individual pixel was then calculated and tabulated. After which, the images depicting the pores of the triangles was transferred and merged using Avizo software (Thermo Scientific, Waltham, MA, USA) to obtain pixels size and areas of triangles. The area of the triangle was obtained using the formula below:

1-pixel area × total pixel of the triangle hole/total number

### 2.5. Mechanical Testing

The mechanical properties assay was conducted in dry environments and the maximum tensile force was analyzed using an EZ-Test machine (Shimadzu, Kyoto, Japan). For this test, we had the samples printed out to be in the shape of a dumbbell and the samples were subsequently stretched from both ends with a rate of 10 mm/min. Six assays were conducted for each sample and the average was recorded.

### 2.6. Cell Viability

In this study, we evaluated biocompatibility using Human Wharton’s Jelly mesenchymal stem cells (WJMSCs, Bioresource Collection and Research Center, Hsin-Chu, Taiwan) that was cultured in a commercially available mesenchymal stem cell medium (#7501, Sciencell, Carlsbad, CA, USA) to passage 4-8. Subsequently, the cells were seeded onto the specimens at a concentration of 104 cells per specimens. The cell viability was determined by the PrestoBlue® (Invitrogen, Grand Island, NY, USA) assay. The values of the absorbance were examined in a multi-well spectrophotometer Infinite M200 PRO (Hitachi, Tokyo, Japan) at 570 nm with a reference wavelength of 600 nm.

### 2.7. Statistical Analysis

Results were subjected to regression analysis. The analysis of variance (ANOVA) method was applied to test the statistical significance of the process parameters. The analysis was carried out at a 9X% confidence level (α = 0.0(10-X). All observations were confirmed by at least three independent experiments. All data are expressed as mean ± standard error (SE).

## 3. Results and Discussion

There are many considerable factors when it comes to designing artificial blood vessels. Previous studies had attempted to fabricate artificial blood vessels that are similar to native blood vessels, but most had failed in attempting to achieve high levels of mimicry [37,38]. Therefore, this study used experimental design methods to optimize printing processes and ratios of materials to attempt to develop artificial blood vessels with good resolution and good biocompatibility. The experimental factors assessed were fabrication process, material formulation, and DLP printing parameters. These mentioned factors were known to affect and have an impact on characteristics of the fabricated artificial blood vessels. In order to improve efficiency and to obtain cost reductions, partial factor experimental designs with three central points were applied with statistical analysis to obtain a significant model. 

### 3.1. Resolution

**Figure 3:**
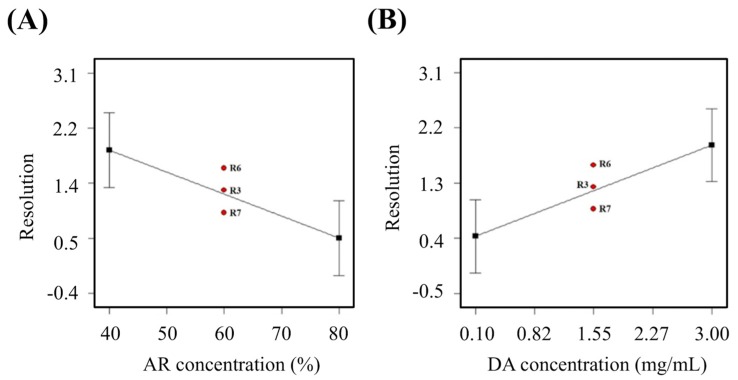
The main effect of (**A**) amine-based resin (AR) concentration and (**B**) dopamine (DA) concentration on the resolution.

The amine-based resin itself is an extremely viscous material and any changes in its concentration ratio would affect printing resolution of the artificial blood vessel. Therefore, difference in AR/HEMA ratio affects the resolution of the material directly. Here, the relationship between ratio of amino resin and resolution was observed. More specifically, it can be seen that the ratio of amino resin and resolution had an inverse proportionate relationship with each other. Printing resolution decreases with increment of amino resin (Figure 3A). In addition, it can be noted that R6, R3, and R7 are in the centre portion of the graph. All three groups had 1.55 mg/mL of dopamine and a curing time of 32 s. However, the resolutions were 1.59 mm^2^, 1.24 mm^2^ and 0.88 mm^2^ respectively. The expected value, or the optimal value was determined to be at 0.975 mm^2^. Therefore, R7 was closest to the expected value. Moreover, it was a well-known fact that addition of DA had also a part to play in affecting the resolution. At this point, it was important to note that DA at the range of 0.1 mg/mL to 3 mg/mL was reported to be able to promote cellular behaviors [39]. In addition, since DA is able to undergo polymerization to form polydopamine, it would be able to act as a light absorber. Thus, appropriate concentration of dopamine can aid in increasing resolutions (Figure 3B). It was also known from our prior studies that differences in DA ratio significantly affects solidification, thus affecting resolutions. Resolutions were proportionate to concentration of DA, thus indicating that higher DA concentrations led to higher resolutions (Table 2). Since amine resin itself is an extremely viscous material, any difference in proportion (ratio of amine resin to hydroxyethyl methacrylate) can drastically affect and influence the physical properties of the printed blood vessel. Lastly, curing durations also had a significant effect on the Young’s modulus. From our prior studies, it can be known that longer the exposure of light led to harder products, thus causing an increment in measured Young’ modulus.

### 3.2. Young’s Modulus

**Figure 4:**
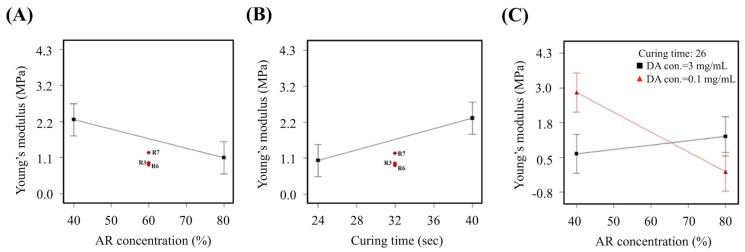
The main effect of (**A**) AR concentration, (**B**) curing time, and (**C**) AR-curing time-DA interaction on the Young’s modulus.

Since the amine-based resin itself is an extremely viscous material, any changes in the proportion of resin would affect hardness and elasticity of the printed blood vessel. The Young’s modulus decreased with increment of resin concentration (Figure 4A). Similarly, it can be noted that R7, R3 and R6 falls in the middle range of the graph. All three groups had 1.55 mg/mL of dopamine and a curing time of 32 s. However, the Young’s modulus was at 1.22 MPa, 0.92 MPa and 0.86 MPa respectively. The expected value, or the optimal value was determined to be between 0.70 MPa to 0.98 MPa and it can be seen that only R3 and R6 falls within the range. On the other hand, the Young’s modulus increased with increment in curing durations (Figure 4B). Therefore, longer duration of light curing led to increased hardness thus translating to increased Young’s modulus. From Figure 4C, we can see an interesting phenomenon of which a low DA concentration of 0.10 mg/mL would lead to a sharp decrement of Young modulus with increment of resin concentration. However, a DA concentration of 3 mg/ml, on the other hand, led to a gradual increment of Young’s modulus with increment of resin concentration. Therefore, it was postulated that increased DA concentration could overcome the viscosity effects of resin, thus making it a more suitable material for tissue engineering.

### 3.3. Cell Viability

**Figure 5:**
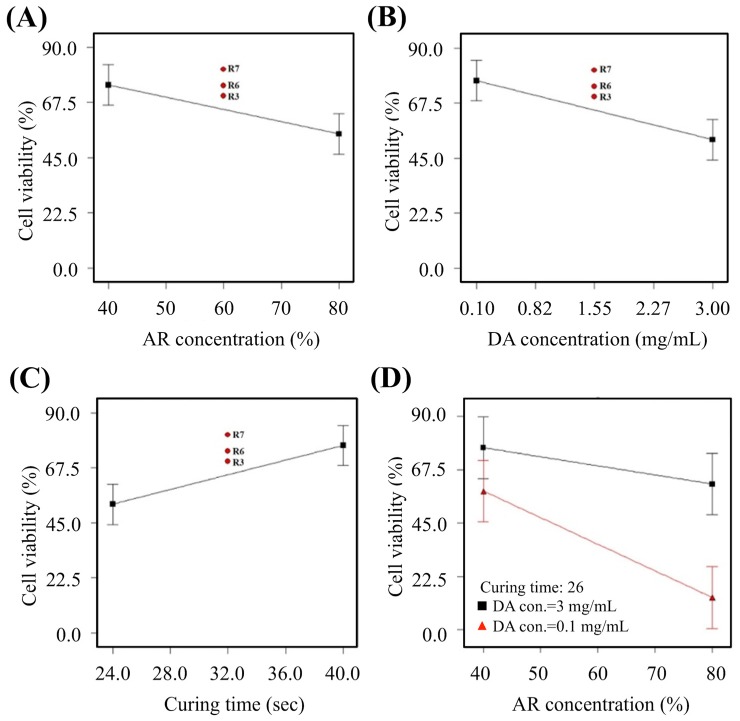
The main effect of (**A**) AR concentration, (**B**) DA concentration, (**C**) curing time, and (**D**) AR-curing time-DA interaction on the cell viability.

Figure 5A showed that increment of resin concentrations led to a gradual decline of cell viability. Similarly, it can be noted that R7, R3, and R6 falls in the middle range of the graph. All three groups had 1.55 mg/mL of dopamine and a curing time of 32 s. However, the cell viabilities were 81.02%, 74.28%, and 70.14% respectively (Figure 5B). In addition, curing duration also had a significant impact on cell viability (Figure 5C). Interestingly, longer duration led to increment of cell viability and together with Figure 5D, our team concluded that increment in DA led to higher photo-curability of material thus having a lesser impact on cells.

### 3.4. Comprehensive Comparison of Experimental Models

**Figure 6:**
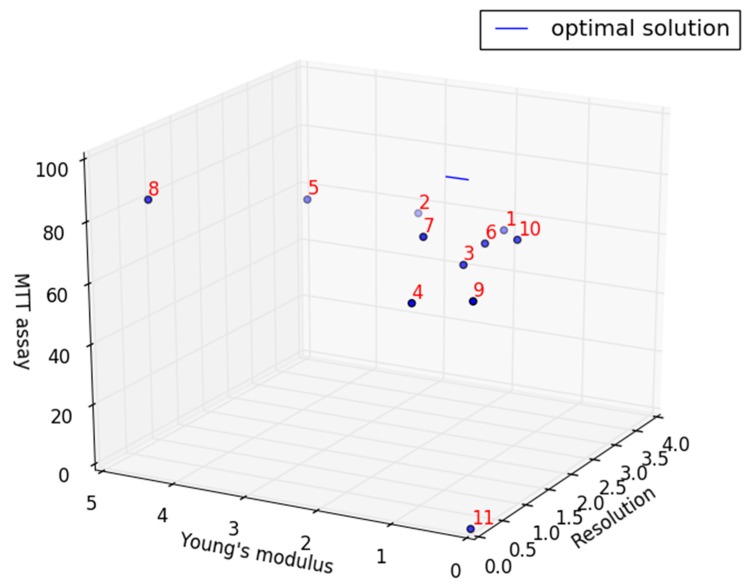
Three-dimensional scatter plot of resolution, Young’s modulus and cell viability.

All experimental samples were then analyzed in a three-dimensional scatter plot as shown in Figure 6 with the optimal properties denoted by a blue line. The optimal properties were determined to be resolution of 0.975 mm^2^, Young’s modulus of 0.701 MPa - 0.983 MPa and highest cellular viability. The formula for this optimal properties were as follows:

$$\begin{array}{l} X=0.975\\ Y=0.282t+0.701,\;0\leq t\leq 1\\ Z=100 \end{array}$$

Table 3 showed the proximity of each group to the optimal properties. Of which, sample 7 and 8 were the nearest to the optimal properties with 18.98 and 10.81 respectively. The experimental parameters of sample 8 were resolution of 0 mm^2^, Young’s modulus of 4.298 MPa and cell viability of 89.23%. In addition, the experimental parameters of sample 7 were resolution of 0.88 mm^2^, Young’s modulus of 1.218 MPa and the cell viability of 81.02 %. Comparing the above two samples, it can be found that the resolution of sample 7 was lower than the expected value of 0.975 mm^2^, thus exhibiting a structure without pores. In addition, its Young’s modulus is larger than the expected value range of 0.701 MPa - 0.983 MPa. On the other hand, the three reaction variables of sample 8 were very close to the expected value, with only the Young’s modulus slightly higher than the expected value. However, this difference was found to be insignificant. Therefore, it can be considered that sample 8 was the closest to native blood vessels.

### 3.5. The Predicted Optimal Factor-level Combination for Verification

It was acknowledged from the prior studies that differences in the ratios of materials would affect properties of materials and the studies showed that addition of DA can improve printing resolutions with DA proportionate to printing resolutions (Table 2). Since the amine resin itself is an extremely viscous material, the difference in proportion (ratio of the amine resin to the hydroxyethyl methacrylate) affects the softness and elasticity of the material. The curing durations also has a significant effect on Young’s modulus. It was known from the prior studies that longer the exposure led to harder product, thus increasing Young’s modulus. Moreover, the statistical analysis of the available rankings, according to the ideal value of the reaction variables (resolution is not 0), the optimal feasible solution is selected/set optimally, and the experimental results are confirmed. After setting (ex: MTT>80% max=100%) the ideal value range, the calculated factors obtained by the calculation (the first one to the fifth group in Table 4) are very similar, so the parameters of the first group (the ratio of amine resin to hydroxyethyl methacrylate is 4:6, DA concentration is 0.1 mg/mL, and the curing time is 26.62 s) were set to be the predicted values.

### 3.6. Optimization Parameter and Fabrication 

**Figure 7:**
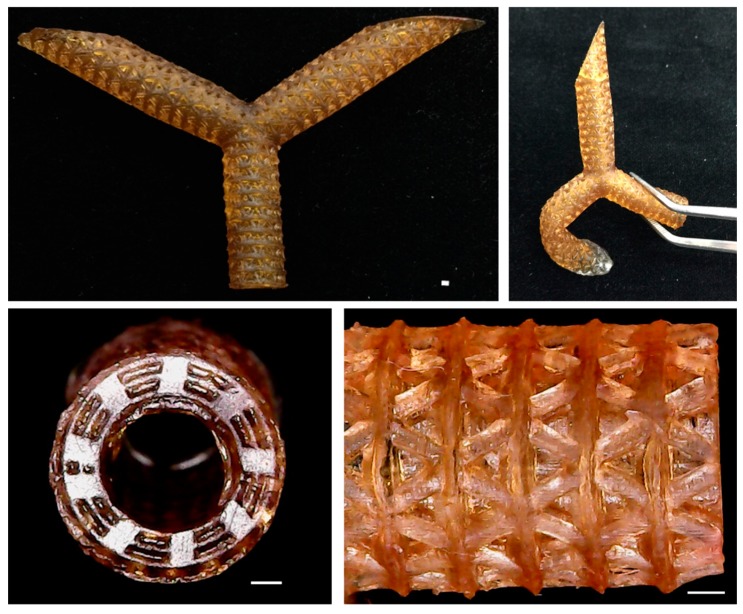
The images of the 3D-printed blood vessels with the optimization parameter. The blood vessels with bi-layer structure and triangle pore. Scale bar is 1 mm.

According to the above parameter, the materials are prepared and printed for analyzed (Figure 7). Compared with the prediction model, the actual value of the resolution is lower than the predicted value, but it is still an acceptable error (Table 5). There are two reasons. One is that the actual value is closer to the true triangle resolution area; the other is that the error value is only -2.68%, which is a small error. The actual value of Young’s modulus is very close to the predicted value, and only 0.55% of the error value shows that the model prediction is very accurate. Finally, the actual value of cell viability is 6.99 % higher than the predicted value, and according to ISO10993, the cell survival rate is more than 80%, which can be regarded as a non-toxic material [40].

## 4. Conclusions

This study explored the significant relationship between different factors and the response variables, and used this to give the optimal parameters for the fabrication of bionic artificial blood vessel stents. This study is based on the use of amine-based resins with soft characteristics that are used as the main material and further modified with dopamine. This material has never been developed in artificial blood vessels and is, therefore, a novel material for artificial blood vessel development. The artificial vascular material developed in this study can be applied to the vascular replacement treatment of coronary artery-related diseases in the future, and the goal of customizing artificial blood vessel stent can be achieved. This AR-based 3D printing photosensitive materials modified with dopamine in different concentrations (S.D.5 w/ dopamine) has been applied to the development of printed scaffolds for blood vessel engineering by applying DLP technology. The modified materials lead to important advantages such as good biocompatibility, which showed 88.37% cell viability, good printing resolution, and the 3D printed scaffolds can facilitate growth of cells. Furthermore, the development may be applied for customized tissue reconstruction in the future.

## Figures and Tables

**Table 1:** Experimental factors and levels.

Experimental Factor	AR con. (%)	DA con. (mg/mL)	Curing time(sec)
Low level	40	0.1	23
High Level	80	3	40

**Table 2:** Experimental factors (columns 2–4) and response variables (columns 5-7) of the 2^3^ fractional factorial screening design (#1–#11).

Run#	AR con. (%)	DA con. (mg/mL)	Curing Time (sec)	Resolution (mm^2^)	Young’s Modulus (MPa)	Cell Viability (%)
1	40.00	3.00	40.00	2.76	1.33	67.26
2	40.00	3.00	24.00	3.05	2.71	65.76
3	60.00	1.55	32.00	1.24	0.92	70.14
4	80.00	0.10	24.00	0.05	0.82	68.64
5	80.00	3.00	40.00	1.86	3.46	77.30
6	60.00	1.55	32.00	1.59	0.86	74.28
7	60.00	1.55	32.00	0.88	1.22	81.02
8	40.00	0.10	40.00	0.00	4.30	89.23
9	80.00	0.10	40.00	0.00	0.00	72.76
10	40.00	0.10	24.00	1.68	0.48	76.06
11	80.00	3.00	24.00	0.00	0.00	0.00

**Table 3:** The distance between the response variables and the coordinate values of the expected values.

Run#	X: Resolution (mm^2^)	Y: Young’s Modulus (MPa)	Z: Cell Viability (%)	Distance	Order
1	2.76	1.333	67.26	32.79	9
2	3.05	2.707	65.76	34.34	10
3	1.24	0.917	70.14	29.86	7
4	0.05	0.822	68.64	31.37	8
5	1.86	3.456	77.30	22.85	3
6	1.59	0.856	74.28	25.73	5
7	0.88	1.218	81.02	18.98	2
8	0.00	4.298	89.23	11.31	1
9	0.00	0.00	72.76	27.26	6
10	1.68	0.484	76.06	23.95	4
11	0.00	0.00	0.00	100	11

**Table 4:** The optimal factor-level combination.

Run#	AR con. (%)	DA con. (mg/mL)	Curing time (sec)	Resolution (mm^2^)	Young’s Modulus (MPa)	Cell Viability (%)
1	40.00	0.10	26.62	1.39	0.98	82.19
2	40.00	0.10	26.54	1.39	0.96	82.12
3	40.00	0.10	26.47	1.40	0.94	82.06
4	41.75	0.10	26.53	1.34	0.98	81.37
5	40.00	0.10	26.74	1.48	0.78	81.47

**Table 5:** Optimization parameter verification.

Parameter	AR con. (%)	DA con. (mg/mL)	Curing Time (sec)	Resolution (mm^2^)	Young’s Modulus (MPa)	Cell Viability (%)
Expectation value	40.00	0.10	26.62	1.39	0.983	82.19
Experimental value	40.00	0.10	26.60	1.35	0.988	88.37
Deviation (%)	0	0	0.75	-2.68	0.55	6.99
